# Interspecific facilitation of micronutrient uptake between cluster-root-bearing trees and non-cluster rooted-shrubs in a *Banksia* woodland

**DOI:** 10.1007/s11104-023-06092-6

**Published:** 2023-06-21

**Authors:** Christiana Staudinger, Michael Renton, Matthias Leopold, Jun Wasaki, Erik J. Veneklaas, Patrícia de Britto Costa, Gustavo Boitt, Hans Lambers

**Affiliations:** 1 School of Biological Sciences, The University of Western Australia, 35 Stirling Highway, Crawley, Perth, 6009 Australia; 2 The ARC Centre of Excellence in Plant Energy Biology, The University of Western Australia, 35 Stirling Highway, Crawley, Perth, 6009 Australia; 3 Institute of Agronomy, Institute of Soil Science, University of Natural Resources and Life Sciences, BOKU Vienna, 3400 Tulln, Austria; 4 Graduate School of Integrated Sciences of Life, Hiroshima University, Higashi-Hiroshima, 739-8521 Japan; 5 School of Agriculture and Environment, The University of Western Australia, 35 Stirling Highway, Crawley, Perth, 6009 Australia; 6 Department of Geography, University of Exeter, Exeter, EX4 4RJ UK

**Keywords:** *Banksia* woodland, Spatial analysis, Cluster roots, Carboxylates, Facilitation, Leaf manganese, Root-root interactions, Size-dependent resource allocation, *Bossiaea eriocarpa*, *Hibbertia hypericoides*

## Abstract

**Background and aims:**

Belowground interspecific plant facilitation is supposed to play a key role in enabling species co-existence in hyperdiverse ecosystems in extremely nutrient-poor, semi-arid habitats, such as *Banksia* woodlands in southwestern-Australia. Manganese (Mn) is readily mobilised by *Banksia* cluster root activity in most soils and accumulates in mature leaves of native Australian plant species without significant remobilisation during leaf senescence. We hypothesised that neighbouring shrubs are facilitated in terms of Mn uptake depending on distance to surrounding cluster root-forming *Banksia* trees.

**Methods:**

We mapped all *Banksia* trees and selected neighbouring shrubs within a study site in Western Australia. Soil samples were collected and analysed for physical properties and nutrient concentrations. To assesses the effect of *Banksia* tree proximity on leaf Mn concentrations [Mn] of non-cluster-rooted woody shrubs, samples of similarly aged leaves were taken. We used multiple linear models to test for factors affecting shrub leaf [Mn].

**Results:**

None of the assessed soil parameters showed a significant correlation with shrub leaf Mn concentrations. However, we observed a significant positive effect of very close *Banksia* trees (2 m) on leaf [Mn] in one of the understorey shrubs. We found additional effects of elevation and shrub size.

**Conclusions:**

Leaf micronutrient concentrations of understorey shrubs were enhanced when growing within 2 m of tall *Banksia* trees. Our model predictions also indicate that belowground facilitation of Mn uptake was shrub size-dependent. We discuss this result in the light of plant water relations and shrub root system architecture.

**Supplementary Information:**

The online version contains supplementary material available at 10.1007/s11104-023-06092-6.

## Introduction

Hyperdiverse plant communities thrive in south-western Australia’s ancient landscapes, which rank among the most nutrient-impoverished habitats worldwide (McArthur 2004; Sauquet et al. 2009; Cook et al. 2015). In highly weathered soils, the majority of phosphorus (P) is present in occluded inorganic or in organic forms (Turner et al. 2018). Plants adapted to nutrient scarcity have evolved a variety of specialized root structures, which can mobilise sparingly available P and micronutrients from the surrounding soil (Lamont 2003; Shane and Lambers 2005). Species of the genus *Banksia* in south-western Australia form compound cluster roots during the wet winter season; cluster roots release carboxylates, protons, phenolic compounds and acid phosphatases into the surrounding soil (Lamont 1982; Lambers et al. 2006). These root structures are confined to the relative P-rich uppermost soil layers (Pate and Watt 2002) and the spatiotemporally concentrated secretion of these root exudates results in the mobilisation of P, manganese (Mn), iron and zinc from sorbed inorganic and organic nutrient pools in the soil (Gardner and Boundy 1983; Grierson and Attiwill 1989; Dinkelaker et al. 1989; Teste et al. 2020).

Several plant species growing on nutrient-poor soils lack specialised root structures capable of acquiring nutrients from poorly-available resource pools (i.e. “nutrient mining”). Nutrient facilitation is considered a plant nutrient-acquisition strategy on highly-weathered soils (Li et al. 2007; Lambers et al. 2022; Shen et al. 2023). Indeed, experiments under controlled conditions indicate that nutrient facilitation may occur among native Australian species that share a long co-evolutionary history (Muler et al. 2014). The non-cluster root forming shrub *Hibbertia racemosa* preferentially allocates root biomass towards the cluster-root forming *Banksia attenuata*, and shows decreased root biomass allocation to the neighbour when grown next to another conspecific (de Britto Costa et al. 2021). Furthermore, imaging of root dynamics in a *Banksia* woodland using minirhizotrons indicated that the likelihood of observing a root of a non-cluster root forming neighbour in the vicinity to a cluster root was substantially greater than observing a non-cluster root next to a non-cluster root (Teste et al. 2020). Taken together, these findings suggest that nutrients mobilised by cluster-root activity of *Banksia* spp. can be shared with non-cluster-root forming neighbours and that interspecific root intermingling with active cluster roots takes place in nutrient-poor soils. However, whether nutrient facilitation occurs in natural habitats still remains to be determined.

In contrast to iron, zinc and copper, plant uptake of Mn is poorly regulated and can therefore result in large variations in leaf Mn concentrations ([Mn]) within a single species (Rengel 2000; Lambers et al. 2021). Roots take up Mn as the divalent cation Mn^2+^. The availability of Mn^2+^ in soils is determined by pH and redox potential of the soil solution. Small decreases in pH can lead to substantial increases in plant-available Mn, especially in moderately acidic soils (Sanders 1983) and wetter, reducing conditions, which can be found in seasonally water-logged soils, can also induce increases in Mn availability (Kirk et al. 2014; Lambers et al. 2021). Pioneer studies in barley and sugarcane grown in solution culture indicate that plant uptake rates of Mn are linearly related to external [Mn] over a wide concentration range of 1–100 µM Mn^2+^ (Maas et al. 1968; Bowen 1981). *Banksia* woodlands are found on highly weathered acidic sandy soils with total [Mn] below 3 mg kg^−1^ (Ritchie et al. 2021) and extremely low exchangeable [Mn] (0.002 mg kg^−1^, values reported for Bassendean sand (Turner and Laliberté 2015; Birnbaum et al. 2018)). As the sandy soils of *Banksia* woodlands are well aerated, reducing conditions due to water-logging are unlikely to occur, even in areas lower in the landscape (Lambers et al. 2021). However, the performance of *Banksia* trees could depend on the access to water from deep sand layers and might therefore be influenced by the elevation at which the trees are growing (Groom 2004; Gao et al. 2020).

In addition to its role as an activator of diverse enzymes (reviewed by Hänsch and Mendel 2009), Mn is involved in photosynthesis in a component of the water-splitting complex of the PSII reaction centre (Schmidt et al. 2016). After root uptake, Mn^2+^ is transported through the xylem to transpiring leaves and shows little remobilisation during leaf senescence due to low phloem mobility (Loneragan 1988). Low remobilisation rates have been shown for temperate woody perennials, crop plants such as *Zea mays*, *Brassica napus* and several native Australian species, including *Banksia* trees and non-cluster root forming neighbouring shrubs (Hayes et al. 2014; Maillard et al. 2015). Given the low soil [Mn] in *Banksia* woodlands, the linear relationship between root uptake rate and external [Mn] (until approximately 100 µM Mn) and the low remobilisation of leaf Mn during leaf senescence, the variation of leaf [Mn] within one plant community can be used as an indicator of rhizosphere modifications such as enhanced exudation of carboxylates (Lambers et al. 2021; Zhou et al. 2022).

Here, our specific aim was to test whether *Banksia* size and proximity to non-cluster rooted woody shrubs influenced leaf [Mn] of non-cluster rooted neighbours. We hypothesised that neighbouring shrubs are facilitated in terms of Mn uptake depending on distance to surrounding cluster-root-forming *Banksia* trees which results in higher leaf [Mn] in shrubs with more and larger nearby *Banksia* trees. For that purpose, we mapped all *Banksia attenuata* and *B. menziesii* trees and selected neighbouring shrubs within a study site located on top of a sand dune in the Swan Coastal Plain in Western Australia (ca. 7800 m^2^). Bulk soil samples (0–7 cm depth) were collected and analysed for physical properties and nutrient concentrations to test for major heterogeneities within the study site. To assesses the effect of *Banksia* tree size and proximity to non-cluster-rooted woody shrubs and shrub elevation on leaf [Mn], samples of similarly-aged leaves were taken, digested and analysed using ICP-OES.

## Materials and methods

### Study area and study site

**Fig. 1:**
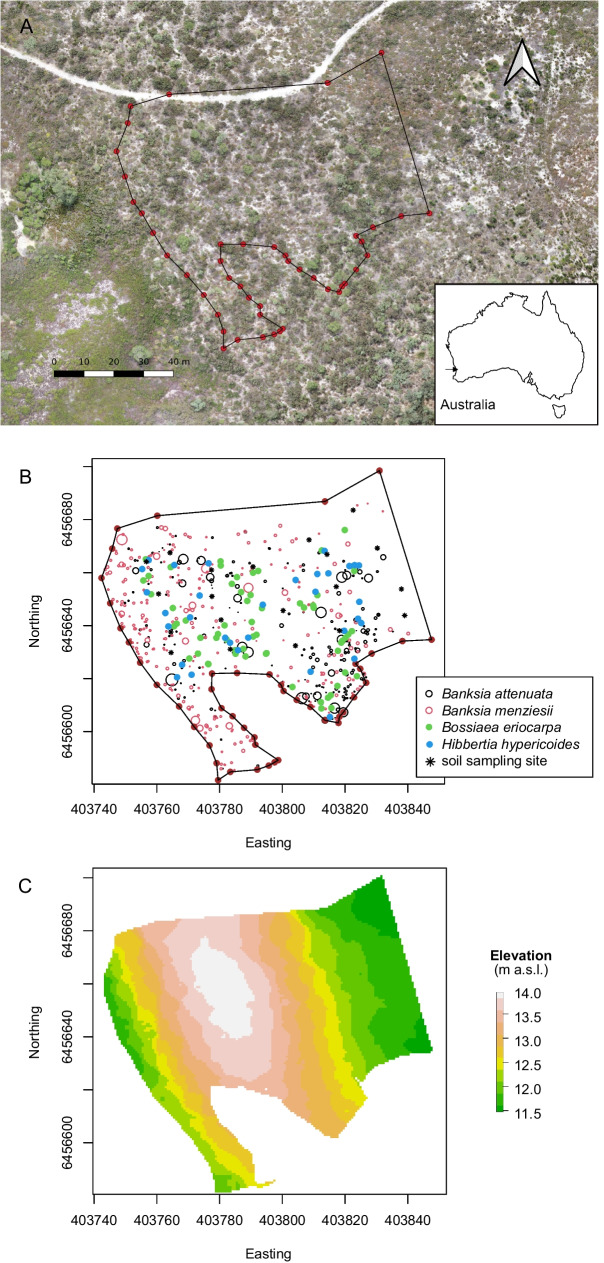
Overview of the Alison Baird Reserve study site. Geographical location of the reserve on the Swan Coastal Plain, Western Australia and boundaries of the study site (7877 m^2^) (**A**). The plant community found on top of a dune is an open sclerophyll *Banksia* woodland with *Banksia attenuata* and *B. menziesii* as the dominant species. Neighbouring understorey woody shrubs within the *Banksia* woodland were chosen randomly and leaves were sampled: *Bossiaea eriocarpa* (*n* = 61), *Hibbertia hypericoides* (*n* = 32). Differences in stem diameter of *Banksia* individuals are indicated by different symbol sizes (**B**). Elevation of the study site(**C**)

The study site was based within Alison Baird Reserve, which is located approximately 15 km southeast of Perth CBD, Western Australia (Baird 1984; Tauss 2019). The reserve is situated on the Swan Coastal Plain, which is a geographic feature composed of marine dune deposits and the Swan River. The reserve comprises an up to 2-million years old dune of the Bassendean sand system (Turner et al. 2018) of about 4 m height whose dune crest is vegetated by *Banksia* woodland dominated by *Banksia attenuata* and *B. menziesii* accompanied by a species-rich community mainly composed of woody shrubs, several perennial herbs and some winter-green herbs (Speck and Baird 1984; Tauss 2019; Smith et al. 2023; for a review on contemporary *Banksia* woodlands see Ritchie et al. (2021)). A site was chosen on the upper part of the dune (7877 m^2^), where *B. attenuata* and *B. menziesii* are the dominating cluster-root bearing species in terms of biomass and where gradients of high and low *Banksia* density and biomass (here termed “*Banksia* effect”) were present (Fig. 1). The study site presented a strongly weathered podsol with acidic humus and a sesquioxide layer at around 5 m. In the topsoil, Colwell-P concentrations are low, typically below 1 mg kg^−1^ (Gao et al. 2020).

### Location mapping, plant species and biomass estimation

Plot boundaries and the positions of *Banksia* trees, neighbouring woody shrubs and soil sampling sites were surveyed using a Trimble R10 GNSS survey system, with base processed to Geoscience Australia AUSPOS (horizontal accuracy 16 ± 4 mm, vertical accuracy 35 ± 6 mm, mean ± SD). Each *Banksia* tree and sapling located within the study site was mapped and individuals of *Bossiaea eriocarpa* Benth. (Fabaceae) and *Hibbertia hypericoides* Benth. (Dilleniaceae) were chosen randomly. Both shrub species showed a large variation of leaf [Mn] in a preliminary screening of various non-cluster rooted species (Wasaki et al., unpublished). Regeneration after fire is mainly achieved through resprouting in all species assessed in this study (Pate and Bell 1999) For plant biomass estimation, *Banksia* tree and sapling stem diameter were measured 1 cm above the lignotuber. Canopy area of *B. eriocarpa* and *H. hypericoides* shrubs were estimated (as a proxy for shrub biomass) using the standard equation for the area of an ellipse A = π*a*b/4, where a and b represent the maximum canopy diameter and the canopy diameter perpendicular to the maximum. Leaf litter cover and depth under *Banksia* canopies was scored at a scale from 0–10. On a plant biomass basis, *B. attenuata* and *B. menziesii* were the dominant species within the study site. Both species form cluster roots which are continuously produced when soil is moist, from May to October, a period during which approximately 80% of the mean annual precipitation falls (Veneklaas and Poot 2003; Lamont 2003; https://weather.agric.wa.gov.au/station/SP). *Banksia* cluster roots have an average lifespan of three weeks (Teste et al. 2018).

### Leaf sampling and elemental composition

Leaf material was collected in late October 2019, during the onset of the dry summer season. For each individual shrub, we collected mature undamaged leaves from the cohort of the previous year which could be visually differentiated based on the differences in lignification of the stem. The leaf material was then oven-dried at 70 °C for 48 h, and ground to a fine powder using a mill and zirconium beads. The ground material was digested using ultra-pure acids (HNO_3_:HClO_4_, 3:1; v/v) and subsequently analysed for P and Mn via inductively coupled plasma optical-emission spectroscopy using Yttrium as an internal standard (Optima 5300DV ICP-OES; PerkinElmer Inc., Waltham, MA, USA).

### Soil sampling, chemical analyses, and texture characterisation

To derive a map of the total soil elemental concentrations, pH and soil particle size distribution within the study site, we collected topsoil samples from 20 locations in late summer, i.e. end of January 2020. Samples were collected from the topsoil after removal of the litter layer, using a bulk density cylinder (7.2 cm diameter, 7.2 cm height, *ca*. 293 cm^3^ inner volume) dried to constant weight at 40 °C and sieved (< 2 mm). Before further analyses, roots with a diameter above 1 mm were removed.

Electrical conductivity (EC) was determined in DI-water suspension (1:5, w/v). Soil pH was determined in DI-water suspension and CaCl_2_ solution (1:5, w/v, 0.01 M CaCl_2_). Total N and C was determined by Dumas dry combustion using an Elementar Vario Macro CNS (Hanau, Germany). Elements in soil were extracted with aqua regia digestion (3:1 mixture of HNO_3_: HCl, v/v, 1 h at 130 °C) and determined by inductively coupled plasma optical emission spectrometry (ICP-OES; 5300DV spectrometer, Perkin Elmer, Shelton, CT, USA) (Rayment and Lyons 2011). Organic matter was removed from an aliquot of the soil samples prior to aqua regia digestion following the method described by (Kunze and Dixon 1986) using hydrogen peroxide according to standard protocols. For the determination of plant available P, soil samples were extracted for 4 h in 0.5 M NaHCO_3_, at pH 8.5 (1:10, w/v, soil solution ratio), phosphate was subsequently determined by the molybdate blue method (Murphy and Riley 1962). The inorganic P in the extracts was analysed by the Dick and Tabatabai (1977) method, the absorbance was recorded at 850 nm (He and Honeycutt 2005).

Particle size distribution of the soil samples was measured by laser diffraction using a Mastersizer 2000 with 200 G wet dispersion accessory (Malvern Panalytical, Malvern, UK). Fractions were split according six fraction sizes from clay to coarse sand at < 2, 63, 200, 630, 2000 μm. No particles with sizes < 2 µm were recorded in the samples analysed.

### Statistical analyses and modelling

Using nearest neighbour interpolation, we obtained estimates for all soil variables for the locations of all shrubs studied. We then tested for correlations between interpolated soil variables and shrub leaf [Mn] using linear regressions.

To test for factors affecting leaf [Mn], we used multiple linear models with leaf [Mn] of the shrub as the dependent variable. In all models we included shrub size (i.e. canopy area) and elevation of the shrub as possible explanatory variables, but we also included different explanatory variables that represented the effect of nearby *Banksia* trees with varying levels of detail. We tried three different modelling approaches for each shrub species. First, we included distance to nearest *Banksia* and size of nearest *Banksia* as possible independent/explanatory variables, with and without transformations of dependent variable. Shrubs less than 5 m from the boundary of our study area were excluded to avoid edge effects, as they may have had nearer *Banksia* trees outside the study area. Second, we explored models with a binary True/False variable representing whether there was any *Banksia* within a certain threshold distance of the shrub. Third, we explored including variables that represented ‘short-range *Banksia* effect’ and ‘long-range *Banksia* effect’. For a given shrub, the short-range *Banksia* effect was defined as the sum of the radii of any *Banksia* trees or saplings within a certain fixed threshold distance independent of the size of the *Banksia*. The long-range *Banksia* effect was defined as the sum of the stem diameters of any *Banksia* trees or saplings within a threshold distance dependent on the size of the *Banksia*; specifically, the threshold distance for this effect was assumed to be a linear function of the stem diameter of the *Banksia* individual. For the second and third approach, we explored different threshold distances and chose distances that optimised the model fit. Shrubs close to the boundary of the study area (close enough that they may have been affected by unrecorded *Banksia* trees beyond the boundaries) were excluded to avoid edge effects. Other *Banksia* effects were also tested, such as an effect declining with increasing distance, but the threshold models explained the data considerably better. The linear models were simplified using stepwise simplification based on AIC (Akaike Information Criteria), to avoid overfitting. All statistical analyses were carried out in R; the analysis code used for modelling is available in the supplements.

## Results

### Alison Baird Reserve study site

We mapped 217 *B. attenuata* and 298 *B. menziesii* trees or saplings using a GNSS survey system with high vertical and horizontal accuracy (Fig. 1). Stem diameters of *B. attenuata* and *B. menziesii* ranged from 5 to 380 mm and 3 to 330 mm within the site (mean ± SE was 75 ± 5 mm and 69 ± 2 mm, respectively). The coordinates of 61 *B. eriocarpa* individuals were recorded and leaves were sampled. A lower number of *H. hypericoides* shrubs were present at the study site. In total, 32 samples were taken of the latter species.

### Leaf [Mn] and shrub canopy area

Within the study site, the leaf [Mn] in one-year old leaves ranged from approximately 27 to 474 mg kg^−1^ in *B. eriocarpa* and from 43 to 287 mg kg^−1^ in *H. hypericoides* (Fig. S1A). We found no significant correlations between leaf [Mn] and leaf [P], neither in *B. eriocarpa* nor in *H. hypericoides* (data not shown).

The canopy area of randomly chosen *B. eriocarpa* individuals ranged from 71 to 5140 cm^2^. We observed a similar range of canopy areas in the 32 *H. hypericoides* individuals (75 to 6704 cm^2^, Fig. S1B).

### Soil properties and litter score

The well-developed podzol was characterised by medium-sized sand as the main textural component, an acidic soil pH and extremely low nutrient concentrations (Table 1). The total soil [Mn] was 6.9 ± 0.6 mg kg^−1^ of which approximately 61 ± 10% was associated with organic matter. Total soil [P] was 13.1 ± 0.7 mg kg^−1^, with 70.3 ± 3.7% in organic form. Bicarbonate extractable soil P was determined spectrophotometrically as an estimate of plant-available P; varying from 1.4 to 4.2 mg kg^−1^.

**Table 1:** Soil properties of the topsoil layer

	unit	mean (SE)	% org. bound
Total C	g kg^−1^	15.0 (1.0)	
Total N	g kg^−1^	1.0 (0.2)	
C/N		24.7 (0.6)	
Ca	mg kg^−1^	506.6 (45)	94 (2)
Fe	mg kg^−1^	477.9 (28)	27 (10)
Al	mg kg^−1^	452.8 (26)	24 (11)
S	mg kg^−1^	79.9 (5.5)	89 (5)
Mg	mg kg^−1^	49.7 (3.6)	61 (10)
Na	mg kg^−1^	47.0 (11)	68 (3)
K	mg kg^−1^	37.5 (14)	46 (3)
P	mg kg^−1^	13.1 (0.7)	70 (4)
P (available)	mg kg^−1^	2.5 (0.16)	
Mn	mg kg^−1^	6.9 (0.6)	61 (10)
pH (DI)		5.5 (0.0)	
pH (CaCl_2_)		4.4 (0.0)	
Water content	wt%	0.2 (0.0)	
Bulk density	g cm^−3^	1.2 (0.0)	
EC	µS cm^−1^	30.2 (2)	
Silt	vol%	0.8 (0.1)	
Fine sand	vol%	2.0 (0.4)	
Medium sand	vol%	64.1 (1.0)	
Coarse sand	vol%	35.1 (1.1)	

Data show the site mean (*n* = 20)

Leaf [Mn] in understorey shrubs did not show a significant correlation with any of the assessed bulk soil parameters (Supp. Table 1). When we performed a regression analysis for *B. eriocarpa* alone, we found a significant correlation between leaf [Mn] and soil pH_(CaCl2)_ (R^2^ = 0.08, F_(1,62)_ = 5.15, *P* < 0.027). However, the correlation between leaf [Mn] of *B. eriocarpa* and soil pH_(DI)_ was not significant (Supp. Table S1). We tested whether the litter score recorded for the nearest *Banksia* affected leaf [Mn] in understorey shrubs and found no significant effect in either shrub.

### Analysis of factors affecting leaf [Mn] of *B. eriocarpa*

**Fig. 2:**
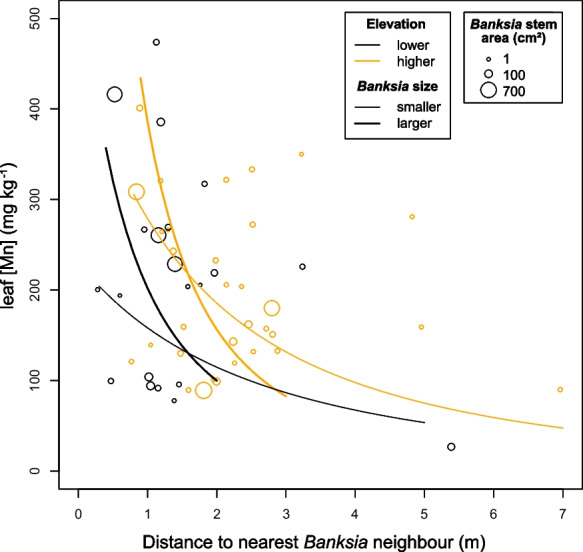
Observations (circles) and model predictions (lines) of leaf [Mn] of *Bossiaea eriocarpa* based on elevation of shrubs, distance to nearest *Banksia* (x-axis), and size of nearest *Banksia* (thick line, larger points: larger *Banksia* stem area, thin lines, smaller points: smaller *Banksia* stem area). Lines are model predictions for *Banksia* trees of size 100 cm^2^ (smaller) and 500 cm^2^ (larger). For values and significance of model coefficients, see Table 2

For *B. eriocarpa*, using our first approach to quantify a potential *Banksia* effect (distance to and size of the nearest *Banksia* individual), we found that leaf [Mn] declined with increasing distance to the nearest *Banksia* tree, increased with greater elevation and was especially high when large *Banksia* trees were within a radius of 2 m (R^2^ = 0.2788, model *P* = 0.003, Table 2, Fig. 2).

**Table 2:** Coefficient estimates and *P*-values for model predicting leaf manganese concentration ([Mn]) of *Bossiaea eriocarpa* with elevation of shrub, distance to nearest *Banksia*, and size of nearest *Banksia* ^a^

Variable	Estimate	*P*-value
Distance	0.00092	0.7600
*Banksia* size	–0.00019	0.0150*
Elevation	0.0096	0.0440*
Distance: *Banksia* size	0.00012	0.0076**

^a^ Overall model F = 4.639, df = 4/48, *P*-value = 0.003, R^2^ = 0.2788. Note that significant interaction indicates leaf [Mn] declined with greater distance to nearest *Banksia* and was especially high when large *Banksia* were within 2 m; see Fig. 2. The model was fitted with a hyperbolic transformation of leaf [Mn] as the dependent variable

Using our third approach, we found several feasible alternative models for *B. eriocarpa* based on short- and long-range *Banksia* effects, along with shrub area and/or elevation which had notably higher R^2^ (R^2^ > 0.6) and lower model *P*-values (*P* < 0.001) (See Supp. Table S2). These models had similar AIC values as the model presented in Fig. 2 and should thus be considered as similarly valid alternative explanations for the data. They were based on fewer data points (due to exclusion of shrubs to avoid edge effects) than the first simpler model and are more difficult to interpret due to significant interactions and correlation between explanatory variables, but, importantly, they all consistently indicate a strong positive effect of larger nearby *Banksia* trees (positive short-range *Banksia* effect). The effect of elevation was again positive, whenever it appeared in these models. These models also suggest that larger shrubs generally had lower leaf [Mn], especially if they did not have nearby *Banksia* trees. For the smaller shrubs, leaf [Mn] appeared to be higher in those shrubs without many *Banksia* trees in their wider vicinity (negative short-range *Banksia* effect for small shrubs). These models also indicated that the largest *Banksia* trees could affect *B. eriocarpa* shrubs up to 14 m away. We did not find a significant model for *B. eriocarpa* using our second approach (*P* = 0.056 for best model found).

### Analysis of factors affecting leaf [Mn] of *H. hypericoides*

**Fig. 3:**
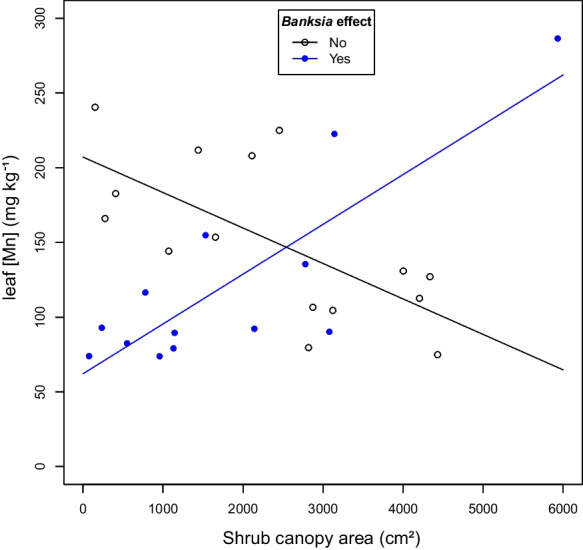
Predictions of leaf Mn concentrations ([Mn]) from best model for *Hibbertia hypericoides*. Predicted (lines) and observed (points) leaf [Mn] for *H. hypericoides* shrubs of different sizes (x-axis) that were or were not affected by *Banksia* neighbours (defined as presence of any *Banksia* within 7.3 m). For values and significance of model coefficients, see Table 3

Using our second approach, we found a good model for *H. hypericoides* using a simple binary variable indicating whether a *Banksia* plant was within a threshold distance of the shrub, where this threshold distance was determined to be 7.3 m (R^2^ = 0.6115, *P* < 0.001, Table 3, Fig. 3). According to this model, small shrubs without *Banksia* individuals within 7.3 m had higher leaf [Mn] than small shrubs with *Banksia* trees within 7.3 m, but as shrub size increased, leaf [Mn] of shrubs without nearby *Banksia* trees decreased and leaf [Mn] of shrubs with *Banksia* trees increased, so that large shrubs without *Banksia* trees within 7.3 m had lower leaf [Mn] than large shrubs with *Banksia* trees within 7.3 m.

**Table 3:** Coefficient estimates and *P*-values for model predicting leaf manganese concentration ([Mn]) of *Hibbertia hypericoides* with area of shrub and simple binary variable indicating whether there was any *Banksia* within 7.3 m as predictors

Variable	Estimate	*P*-value
Affected	–145	< 0.0001 ***
Shrub area	–0.0237	0.0024 **
Short-range *Banksia* effect	1.92	0.0029 **
Affected:Shrub area	0.0571	< 0.0001 ***

^*^ Overall model F = 12.59, df = 3/24, *P*-value < 0.0001, R^2^ = 0.6115. Note that significant interaction indicates leaf [Mn] tended to increase with shrub area for shrubs that have *Banksia* trees within 7.3 m and decrease with shrub area for shrubs that do not have any *Banksia* tree within 7.3 m; see Fig. 3

Using our third approach, we also found an alternative model for *H. hypericoides* based on short- and long-range *Banksia* effects which indicated similar patterns to the simpler binary variable model, also with a threshold distance of 7.3 m (long-range effect) (see Supp. Table S3). However, this model indicated that leaf [Mn] increased with shrub size even more steeply when there was a *Banksia* tree within 1 m (short-range effect). Both models for *H. hypericoides* showed similar patterns to the alternative models for *B. eriocarpa,* based on short- and long-range *Banksia* effects (Supp. Table S2). We did not find a significant model for *H. hypericoides* using our first approach (*P* = 0.12 for best model found).

## Discussion

Belowground facilitation among plant species with different nutrient-acquisition strategies has been proposed as a mechanism that contributes to maintenance of hyperdiverse plant communities on highly-weathered soils (Lambers et al. 2015; Teste et al. 2017). Cluster-root activity increases Mn uptake, as well as leaf [Mn] in Proteaceae, including *Banksia* (Lambers et al. 2021). Neighbouring species without cluster roots show root intermingling and enhance nutrient uptake when grown next to *Banksia* plants in greenhouse experiments (Muler et al. 2014; de Britto Costa et al. 2021). In field surveys, species of the non-cluster root-forming genera *Bossiaea* and *Hibbertia* interestingly present high leaf [Mn], relative to other non-cluster-rooted species (Abrahão et al. 2018; Zhong et al. 2021; Lambers et al. 2022). However, whether the distance to *Banksia* trees explains variation in leaf [Mn] of neighbouring shrubs growing in natural environments has not been explored so far. We found evidence for higher leaf [Mn] in *B. eriocarpa* shrubs, when *Banksia* plants with a larger stem diameter were growing in proximity relative to shrubs that did not grow close to *Banksia* trees (Fig. 2). However, based on empirical data, our models also predict additional significant effects of elevation on leaf [Mn] in *B. eriocarpa* and of shrub biomass in both *B. eriocarpa* and *H. hypericoides* (estimated by shrub canopy size, Supp. Table S2, Fig. 3).

### Variation in leaf [Mn] in two understorey shrub species

In both sympatric species, *B. eriocarpa* and *H. hypericoides*, we found a large variation in [Mn] of ~ 1-year old leaves which was not related to bulk soil [Mn] or any other topsoil parameters assessed, suggesting that the occurrence of a *Banksia* effect could be tested with these two species. Within our study site, we observed the highest leaf [Mn] in *B. eriocarpa* (> 400 mg kg^−1^), while maximum values in *H. hypericoides* were below 300 mg kg^−1^. Both shrub species share similar flowering phenology and responses to fire-prone environments, but differences in root system architecture have been reported (Dodd et al. 1984; Pate and Bell 1999; Veneklaas and Poot 2003; Clarke et al. 2015). While *H. hypericoides* produces a shallow main root with several lateral roots in the uppermost soil layers (Pate and Bell 1999), *B. eriocarpa* presents fewer lateral roots and produces a deeper taproot reaching ~ 2 m in depth (Dodd et al. 1984). A detailed analysis of water relations in a *Banksia* woodland showed that shrubs with deeper root systems present higher stomatal conductance during the onset of drier summer months, whereas shrubs with shallower root systems reduce stomatal conductance as early as late spring (Veneklaas and Poot 2003). If the deeper taproot of *B. eriocarpa* allowed the shrubs to access water for a longer time throughout the year, it is conceivable that faster cumulative transpiration rates contributed to the higher [Mn] of mature leaves relative to the leaves of shallow-rooted *H. hypericoides* shrubs.

### The effect of elevation on leaf [Mn] in *B. eriocarpa*

To test whether the large variation in leaf [Mn] might be explained by a *Banksia* effect, we used a modelling approach using elevation, shrub canopy area and different definitions of a potential *Banksia* effect as explanatory variables. For *B. eriocarpa*, leaf [Mn] was higher on top of the dune, particularly when larger *Banksia* trees were present within 2 m. This positive effect of elevation could be related to differences in the extent of cluster-root formation and activity, as *B. attenuata* and *B. menziesii* depend on water uptake from deep sand layers (> 4 m depth), which are not present at the lower parts of the dune (Groom 2004; Gao et al. 2020). *B. eriocarpa* forms fewer lateral roots than *H. hypericoides* (Pate and Bell 1999) and might, therefore, be more sensitive to variation in *Banksia* performance.

### *Banksia* effects and interactions with shrub biomass on leaf [Mn]

Our data show that leaf [Mn] was higher in *B. eriocarpa* shrubs that had very close *Banksia* neighbours than in conspecifics without *Banksia* individuals within approximately 2 m of the shrub’s base, partially confirming our initial hypothesis (Fig. 2).

In addition to the positive effect of close *Banksia* trees on shrub leaf [Mn], we found an interaction of *Banksia* effect with shrub canopy area in the more complex models for *B. eriocarpa* and in a simpler model for *H. hypericoides* (Supp. Table S2, Fig. 3). Larger shrubs had lower leaf [Mn] when there were no nearby *Banksia* trees present, when compared with larger shrubs growing close to *Banksia* individuals; and smaller shrubs had higher leaf [Mn] when no *Banksias* trees were present nearby. This result indicates that complex interactions exist between plant size and accumulation of Mn that tends to follow the transpiration stream.

If cluster-root activity facilitates Mn uptake in neighbouring shrubs, why are leaf [Mn] higher in small shrubs growing in areas with low *Banksia* density relative to small shrubs grown in areas with a high *Banksia* effect? One possible explanation is that the presence of *Banksia* neighbours alters root biomass accumulation at greater soil depth, as well as root system architecture. Neighbour effects on root-allocation patterns have been reported previously (Gersani et al. 2001; Li et al. 2006; de Britto Costa et al. 2021). In a semiarid ecosystem, such changes in root system architecture might result in relatively shorter or longer periods of transpiration throughout the year. Accordingly, small shrubs growing away from *Banksia* trees would produce deeper main roots, have faster annual cumulative transpiration rates and therefore accumulate more Mn in their leaves. Small shrubs growing among *Banksia* individuals could produce more lateral roots and shorter main roots. When grown in rhizoboxes, *H. racemosa* allocated more root biomass towards the roots of a *B. attenuata* neighbour than towards the roots of a conspecific (de Britto Costa et al. 2021). During the dry season, *Banksia* trees exhibit hydraulic lift of water from deeper to more shallow soil horizons (Burgess et al. 2000) which might influence rooting patterns in neighbouring shrubs. However, the idea of differential rooting depths of understorey shrubs in response to *Banksia* neighbours requires further investigation. Root allocation, together with seasonal patterns in plant water relations of shrubs growing close to or away from *Banksia* trees have to be assessed in the future.

In conclusion, our study indicates that Mn uptake of understorey shrubs can be facilitated by very close *Banksia* trees, especially if they are tall. Our model predictions reveal that complex interactions between *Banksia* effect and neighbouring shrub size exist which might be related to the rooting depths of understorey shrubs resulting in different annual transpiration rates and ultimately leaf [Mn].

### Supplementary Information

Below is the link to the electronic supplementary material.

Supplementary file1 (PDF 443 KB)

Supplementary file2 (ZIP 415 KB)

## Data Availability

The datasets generated and analysed during the current study and the R code used for modeling are available in the supplements.

## Abbreviations

ICP-OES: Inductively Coupled Plasma – Optical Emission Spectroscopy
[Mn]: Manganese concentration(s)
SRBE: Short-range *Banksia* effect
LRBE: Long-range *Banksia* effect
