# Supplementary material for: Interspecific facilitation of micronutrient uptake between cluster-root-bearing trees and non-cluster rooted-shrubs in a Banksia woodland
Source: Plant Soil. 2023 Jun 21;496(1-2):71–82. doi: 10.1007/s11104-023-06092-6 (PMC10948572; doi:10.1007/s11104-023-06092-6)
Supplement: Supplementary file 1 — Supplementary file1 (PDF 443 KB) [file 11104_2023_6092_MOESM1_ESM.pdf]

Supporting Information

Title: Interspecific facilitation of micronutrient uptake between cluster-root bearing trees and non-cluster rooted shrubs in a *Banksia* woodland

Journal: Plant and Soil

Authors: Christiana Staudinger, Michael Renton, Matthias Leopold, Jun Wasaki, Erik J. Veneklaas, Patrícia de Britto Costa, Gustavo Boitt and Hans Lambers

* Corresponding author: christiana.staudinger@boku.ac.at, University of Natural Resources and Life Sciences, BOKU Vienna, 3400 Tulln, Austria

Table S1 Summary of linear regression models relating shrub leaf Mn concentrations to interpolated soil variables.

Summary of linear regression models relating *shrub* leaf Mn concentrations and interpolated soil variables

soil variable	R^2	F	P	slope
water content	0.00043555	0.042	0.838	-75.365
bluk density	0.0026596	0.259	0.612	102.514
cluster root score	0.00106944	0.104	0.748	9.645
electric conductivity	0.00025868	0.025	0.874	-0.382
pH(DI)	0.00470397	0.458	0.5	86.089
pH($CaCl_2$)	0.02371062	2.356	0.128	-290.744
Silt	0.01009349	0.989	0.322	-35.049
Fine sand	0.02650092	2.641	0.107	-20.965
Medium sand	0.00271438	0.264	0.609	2.652
Coarse sand	0.00145156	0.141	0.708	-1.973
N	0.00624638	0.61	0.437	-1299.593
C	0.00647281	0.632	0.429	-44.789
C:N	0.00025981	0.025	0.874	-1.779
Al	0.0337257	3.386	0.069	-0.409
Ca	0.01772137	1.75	0.189	-0.17
Fe	0.01806287	1.784	0.185	-0.344
K	0.00907351	0.888	0.348	-1.453
Mg	0.01243049	1.221	0.272	-1.911
Mn	0.0261591	2.606	0.11	-15.711
Na	0.01256734	1.235	0.269	-0.652
P	0.02902461	2.9	0.092	-10.625
P (available)	0.029025	2.899545	0.091807	-10.6246
S	0.01106588	1.085	0.3	-1.091

Summary of linear regression models relating *Bossiaea eriocarpa* leaf Mn concentrations and interpolated soil variables

soil variable	R2	F	P	slope
water content	0.00154252	0.096	0.758	-144.669
bluk density	0.00042469	0.026	0.872	-41.173
cluster root score	0.00554142	0.345	0.559	23.398
electric conductivity	0.0090145	0.564	0.456	2.375
pH(DI)	0.00024098	0.015	0.903	18.563
pH(CaCl2)	0.07672223	5.152	0.027	-568.911
Silt	0.0311781	1.995	0.163	-65.281
Fine sand	0.05052855	3.299	0.074	-29.897
Medium sand	0.00498089	0.31	0.579	-3.829
Coarse sand	0.00982384	0.615	0.436	5.524
N	0.00012712	0.008	0.93	205.801
C	1.1556E-05	0.001	0.979	2.15
C:N	0.00097643	0.061	0.806	3.607
Al	0.0329452	2.112	0.151	-0.438
Ca	0.00224999	0.14	0.71	-0.069
Fe	0.0056387	0.352	0.555	-0.209
K	0.00024185	0.015	0.903	0.259
Mg	6.4881E-07	0	0.995	-0.016
Mn	0.05060272	3.305	0.074	-22.747
Na	0.00010958	0.007	0.935	-0.072
P	0.02408819	1.53	0.221	-10.812
P (available)	0.033661	2.159649	0.146733	-37.0703
S	0.00027207	0.017	0.897	-0.194

Summary of linear regression models relating *Hibbertia hypericoides* leaf Mn concentrations and interpolated soil variables

soil variable	R^2	F	P	slope
water content	0.01938364	0.652	0.425	441.056
bluk density	0.01672449	0.561	0.459	228.848
cluster root score	0.02980533	1.014	0.321	43.927
electric conductivity	0.0221858	0.749	0.393	-2.899
pH(DI)	0.03798438	1.303	0.262	242.639
pH(CaCl2)	0.00491586	0.163	0.689	-105.095
Silt	1.6085E-06	0	0.994	0.356
Fine sand	0.0086297	0.287	0.596	-10.022
Medium sand	0.02453723	0.83	0.369	6.585
Coarse sand	0.02494551	0.844	0.365	-6.687
N	0.01057019	0.353	0.557	-1332.313
C	0.00495346	0.164	0.688	-30.4
C:N	0.01334244	0.446	0.509	11.481
Al	0.03526781	1.206	0.28	-0.327
Ca	0.02787989	0.946	0.338	-0.163
Fe	0.05652486	1.977	0.169	-0.473
K	0.02461631	0.833	0.368	-1.937
Mg	0.01893041	0.637	0.431	-1.76
Mn	0.01862454	0.626	0.434	-11.062
Na	0.01624259	0.545	0.466	-0.556
P	0.02344353	0.792	0.38	-7.236
P (available)	0.002139	0.070728	0.791935	-8.3724
S	0.01653837	0.555	0.462	-1.026

Table S2: Coefficient estimates and *P*-values for alternative models predicting leaf Mn concentrations ([Mn]) of *Bossiaea eriocarpa* with area of shrub, elevation, long-range *Banksia* effect (<14m, LRBE), short-range *Banksia* effect (<1.6 m, SRBE) and a binary variable representing whether there were any *Banksia* individuals within 14 m (AnyBk) as predictors. *

	**Model 1**		**Model 2**		**Model 3**		**Model 4**	
	Estimate	*P*	Estimate	*P*	Estimate	*P*	Estimate	*P*
(Intercept)	471.3955	0.356082	404.0383	<0.0001	-419.771	0.214294	519.9635	0.318143
SRBE	1.042873	0.003296	0.768106	0.007046	1.168234	0.001129	1.270255	0.010879
Shrub area	-	-	-0.05919	0.009131	-0.02399	0.084598	-0.02741	0.040306
log(Shrub area)	-89.2376	0.034575	-	-	-	-	-	-
LRBE	-1.31129	0.011837	-0.44583	<0.0001	-0.30121	<0.0001	-0.26698	<0.0001
eff2:Shrub Area	-	-	0.000136	0.022863	-	-	-	-
LRBE:log(Shrub area)	0.146179	0.040157	-	-	-	-	-	-
AnyBk	-	-	-	-	-		-1568.75	0.027807
Elevation	36.02812	0.176813	-	-	58.02039	0.028058	-12.0731	0.754095
AnyBk:Elevation	-	-	-	-	-	-	117.6021	0.026995
model F	8.8		10.49		10.24		8.9	
model df	5/22		4/23		4/23		6/21	
model p-value	0.0001		<0.0001		<0.0001		<0.0001	
R^2	0.67		0.65		0.64		0.72	
AIC	318.2		318.0		318.4		315.7	

* For all these models, 1.6 m was the fixed SRBE threshold distance for any sized *Banksia* individual and 14 m was the threshold distance for the largest *Banksia* tree for the LRBE threshold distance that varied linearly with *Banksia* radius. Note that significant interactions between LRBE and shrub area indicate leaf [Mn] tended to increase with shrub area for shrubs that have many larger *Banksia* trees within 14 m and decrease with shrub area for shrubs that do not have many larger *Banksia* trees within 14 m.

Table S3: Coefficient estimates and *P*-values for alternative model predicting leaf [Mn] of ***Hibbertia hypericoides*** with area of shrub, elevation, long-range *Banksia* effect (<7.3 m, LRBE), short-range *Banksia* effect (<1 m, SRBE) as predictors. *

	Estimate	*P*
(Intercept)	-223.929	0.163586
SRBE	-31.0839	0.046187
Shrub area	-0.02195	0.006531
LRBE	-0.35788	<0.0001
Elevation	33.0104	0.014885
SRBE: Shrub area	0.010191	0.041715
LRBE: Shrub area	0.000147	0.000175
model F	6.2	
model df	6/21	
model p-value	0.0007	
R^2	0.64	

* For this model, 1 m was the fixed SRBE threshold distance for any sized *Banksia* individual and 7.3 m was the threshold distance for the largest *Banksia* tree for the LRBE threshold distance that varied linearly with *Banksia* tree radius. Note that significant interactions between LRBE and shrub area indicate leaf [Mn] tended to increase with shrub area for shrubs that have many larger banksias within 7.3m and decrease with shrub area for shrubs that do not have many larger *Banksia* trees within 7.3m.

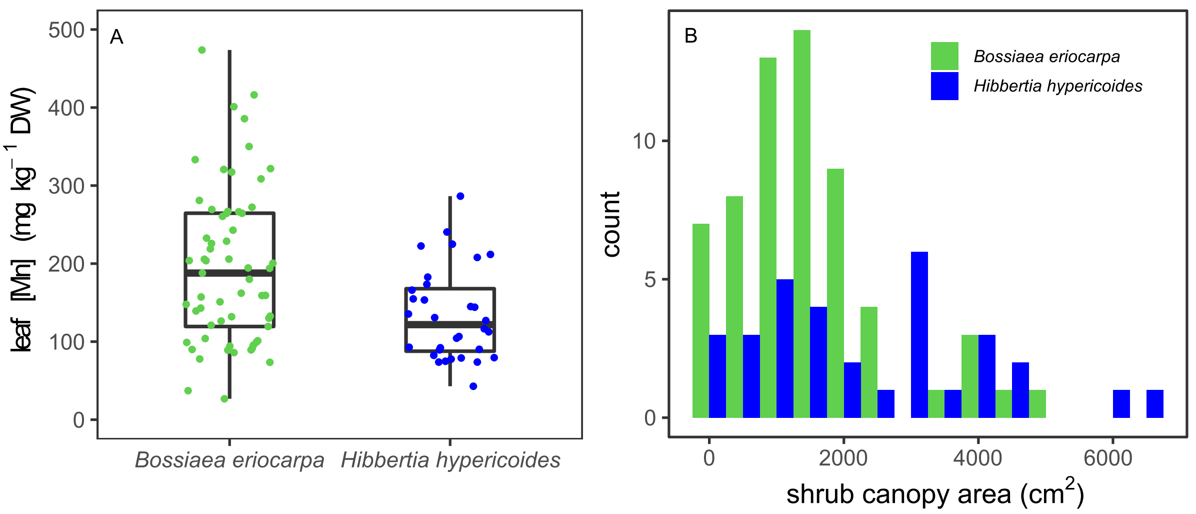

Fig. S1 | Variation in leaf manganese concentration ([Mn]) and distribution of shrub canopy area within the study site. Variation in leaf [Mn] of the previous year's leaves of understorey woody shrubs within the *Banksia* woodland study site (A). Histogram of shrub canopy area of randomly selected individuals sampled in this study (B). *Bossiaea eriocarpa* (n = 61), *Hibbertia hypericoides* (n = 32).
